# Hepatoprotective effects of magnolol in fatty liver hemorrhagic syndrome hens through shaping gut microbiota and tryptophan metabolic profile

**DOI:** 10.1186/s40104-024-01074-9

**Published:** 2024-09-06

**Authors:** Yujie Lv, Chaoyue Ge, Lianchi Wu, Zhaoying Hu, Xinyu Luo, Weichen Huang, Shenao Zhan, Xinyu Shen, Dongyou Yu, Bing Liu

**Affiliations:** 1 Hainan Institute, Zhejiang University, 572000 Sanya, China; 2 College of Animal Sciences, Zhejiang University, Hangzhou, 310058 China

**Keywords:** Fecal microbiota transplantation, FLHS, Gut–liver axis, Laying hens, Magnolol

## Abstract

**Background:**

Magnolol (MAG) exhibits hepatoprotective activity, however, whether and how MAG regulates the gut microbiota to alleviate fatty liver hemorrhagic syndrome (FLHS) remains unclear. Therefore, we investigated the mechanism of MAG in FLHS laying hens with an emphasis on alterations in the gut–liver axis. We randomly divided 540 56-week-old Hy-line white laying hens with FLSH into 4 groups. The birds were fed a high-fat low-protein (HFLP) diet (CON) or HELP diets supplemented with 200, 400, and 600 mg/kg of MAG (M1, M2, and M3, respectively) for 9 weeks.

**Results:**

Magnolol supplementation increased the laying rate and ameliorated hepatic damage and dysfunction by regulating lipid metabolism, improving intestinal barrier function, and shaping the gut microbiota and tryptophan metabolic profiles. Dietary MAG supplementation downregulated the expression of lipid synthesis genes and upregulated the expression of lipid transport genes at varying degrees. The intestinal barrier function was improved by 200 and 400 mg/kg of MAG supplementation, as evidenced by the increased villus height and mRNA expression of tight junction related genes. Microbiological profile information revealed that MAG changed the gut microbiota, especially by elevating the abundances of *Lactobacillus, Faecalibacterium*, and *Butyricicoccus*. Moreover, non-targeted metabolomic analysis showed that MAG significantly promoted tryptophan metabolites, which was positively correlated with the MAG-enriched gut microbiota. The increased tryptophan metabolites could activate aryl hydrocarbon receptor (*AhR*) and relieved hepatic inflammation and immune response evidenced by the downregulated the gene expression levels of pro-inflammatory cytokines such as interleukin-1β (*IL-1β*), tumor necrosis factor-α (*TNF-α*), and interleukin-6 (*IL-6*) in the liver. The fecal microbiota transplantation (FMT) experiments further confirmed that the hepatoprotective effect is likely mediated by MAG-altered gut microbiota and their metabolites.

**Conclusions:**

Magnolol can be an outstanding supplement for the prevention and mitigation of FLHS in laying hens by positively regulating lipid synthesis and transport metabolism, improving the intestinal barrier function, and relieving hepatic inflammation by reshaping the gut microbiota and metabolite profiles through gut microbiota-indole metabolite-hepatic AhR crosstalk. These findings elucidate the mechanisms by which MAG alleviates FLHS and provide a promising method for preventing liver diseases by modulating gut microbiota and their metabolites.

**Supplementary Information:**

The online version contains supplementary material available at 10.1186/s40104-024-01074-9.

## Background

As laying hens gradually enter the later laying stages, their production performance and egg quality begin to decline [1]. Notably, the livers of laying hens undergo further hepatic lipid deposition with varying degrees of aging, which in turn results in a certain degree of damage, even to the extent of fatty liver hemorrhagic syndrome (FLHS). FLHS is distinguished by the pathological accumulation of hepatic and abdominal lipids, manifesting as hepatic steatosis, liver rupture, inflammatory reactions, and hemorrhagic episodes within the liver [2, 3], which reduce egg production, fertilization rate, and hatchability [4], thus affecting economic benefits. The pathogenesis of FLHS arises from an imbalance in lipid metabolism triggered by diverse factors including nutrition, metabolism, environmental conditions, hormonal influences, and genetics. The underlying causes of fatty liver disease closely resemble those of FLHS [5]. Therefore, to ensure the durability of egg production and the stability of egg quality during the laying cycle, it is essential to explore ways to mitigate liver injury in FLHS laying hens.

In recent years, more researchers have focused on plant polyphenols, and the main reason for this interest is the possible role of polyphenols in the preventive aspects of a variety of diseases associated with oxidative stress (e.g., cancer and cardiovascular diseases) [6–8] as well as lipid metabolism disorders (e.g., hepatocellular carcinoma) [9]. Among them, *Magnolia officinalis* has a wide range of pharmacological effects, including regulation of gastrointestinal function, anti-pathogenic microorganisms, and anti-inflammatory effects. The chemical composition of *Magnolia officinalis* includes phenols, alkaloids and volatile oils. After a thorough isolation and identification process, over 200 distinct compounds have been discovered and characterized [10]. Of these compounds, magnolol (MAG), honokiol, 4-methoxyhonokiol, and other major components have attracted considerable attention for their outstanding activities. Studies have shown that honokiol exerts protective effects counteracting oleic acid-induced liver injury and tyloxapol-induced hyperlipidemia in mice [11]. Kim et al. [12] found that honokiol improves insulin resistance and fatty liver metaplasia in mice with type 2 diabetes. The administration of MAG to mice on a high-fat diet inhibited lipogenesis, resulting in a reduction in the size of adipocytes, and mRNA expression of key genes responsible for the synthesis and uptake of fatty acids, such as fatty acid synthase (*FAS*), acetyl-CoA carboxylase (*ACCOX*) and stearoyl-CoA desaturase (*SCD*) were also reduced [13]. MAG has recently gained attention as a functional additive in poultry nutrition for improving liver health.

The mechanism by which MAG alleviates liver injury mainly involves the regulation of the abundance of intestinal microbiota [14] and its metabolites [15]. The intestinal microbiota is involved in the isomerization of MAG during its absorption and metabolism in vivo [16], the abundance of which is also influenced by MAG. The main effect MAG on the intestinal microbiota is to reduce detrimental bacteria and increase beneficial microbiota components [17]. Supplementation with MAG increases the abundance of *Lactobacillus *genus and decreases *Streptococcus *genus, thus regulating metabolic pathways to resist *Salmonella pullorum*-induced intestinal barrier damage [18].

FLHS is reportedly associated with gut microbiota dysbiosis with alterations in the abundance of Firmicutes and Bacteroidota and their metabolites, which exhibit a strong correlation with the extent of fibrosis and nonalcoholic steatohepatitis [19, 20]. Whether and how MAG regulates the intestinal microbiota and the specific metabolites that subsequently affect FLHS remains uncertain. In this study, the aged laying hens with FLHS induced by a high-fat low-protein (HFLP) diet were used to determine the regulatory mechanism underlying whether and how MAG regulates the intestinal microbiota and their metabolites to alleviate the liver-associated metabolic disorders of FLHS. The findings of this study can provide a theoretical basis and research foundation for the application of MAG to alleviate FLHS in laying hens.

## Methods

### Animal experimental design, management, and diet

In order to establish the FLHS model, a total of 1,000 Hy-line white laying hens aged 46 weeks were fed a HFLP diet. After 10-week HFLP diet treatment, the blood tests were performed, and a total of 540 56-week-old laying hens with liver-associated damage and lipid metabolic disorders were selected and randomly divided into 4 groups, with 9 replicates in each group and 15 hens in each replicate. Hens in the four groups were fed the HFLP diet (negative control, CON) and HFLP diets supplemented with 200, 400, and 600 mg/kg of MAG (M1, M2, and M3, respectively) for 9 weeks. Magnolol was purchased from Beijing Huawei Ruike Chemical Technology Co., Ltd. (Beijing, China), which was obtained from the bark of *Magnolia officinalis* with 80% purity. The composition and nutrient levels of the basal and HFLP diets is shown in Table 1. Birds were kept in staggered three-layer cages. The indoor was maintained at 26 °C, while the humidity level was sustained at 65%, ensuring a comfortable and controlled environment within the house. The animal experiment protocol and procedures of this study have been approved by the Animal Care and Use Committee of Zhejiang University (Hangzhou, China, Protocol number ZJU20220310) and implemented in accordance with the relevant guidelines and regulations.

**Table 1:** Ingredients compositions and nutrient levels of the experimental diets

Items	Basal diet	HFLP diet^a^
Ingredients, %		
Corn	56.00	61.00
Soybean meal (44% CP)	25.50	15.00
Wheat middling (14.5% CP)	4.00	4.00
Emulsified fat powder (50% Fat)	2.00	-
Lard	-	7.00
Limestone	9.00	9.00
CaHPO_4_	1.00	1.10
Salt	0.30	0.30
DL-Methionine	0.20	0.25
L-Lysine HCl (78%)	-	0.35
Phytase	0.03	0.03
Choline chloride	0.12	0.12
Premix^b^	1.85	1.85
Total	100	100
Nutrient levels^c^		
Metabolic energy, Mcal/kg	2.65	3.10
Crude protein, %	16.48	12.50
Ca, %	3.51	3.50
Available P, %	0.35	0.36
Met, %	0.46	0.46
Lys, %	0.84	0.84

^a^HFLP diet: High-fat low-protein diet

^b^The premix provided the following per kg of the diet: vitamin A 12,500 IU, vitamin D_3_ 4,000 IU, vitamin E 80 IU, vitamin K_3_ 2 mg, vitamin B_12_ 5 mg, thiamine 1 mg, riboflavin 8.5 mg, calcium pantothenate 50 mg, niacin acid 32.5 mg, pyridoxine 8 mg, folic acid 5 mg, iron 60 mg, copper 10 mg, manganese 80 mg, zinc 80 mg, iodine 0.3 mg, selenium 0.30 mg, and antioxidant 2.00 mg

^c^Nutrient levels were calculated values except for crude protein and Ca contents

### Sample collection and preparation

At the end of the 9-week trial period, one laying hen per replicate with a body weight close to the mean was selected. After fasting for 12 h, the venous blood was collected to separate serum by centrifuging at 4 °C at 3,000 × *g* for 10 min. Then the hens were euthanized and the whole liver and abdominal adipose tissues were excised and weighed to calculate organ indexes. A portion of the liver tissue and the middle part of the jejunum were fixed in 4% formaldehyde or 2.5% glutaraldehyde solution, while the remaining liver and jejunum tissue were frozen for further analysis. The cecal contents were obtained and immediately snap frozen in liquid nitrogen and stored at –80 °C for further analysis.

### Laying performance

Daily records of feed intake, egg production, and egg weight were obtained, and laying rate (LR), average daily feed intake (ADFI), average daily egg mass (ADEM) and feed conversion rate (FCR) were calculated according to the methods described by Liu et al. [21].

### Serum biochemical parameters

The activity of aspartate transferase (AST) and alanine transferase (ALT) activity as well as the levels of triglyceride (TG), total cholesterol (TC), high-density lipoprotein cholesterol (HDL-C), and low-density lipoprotein cholesterol (LDL-C) in serum were measured using an automatic biochemical analyzer (Hitachi 7600; Hitachi Hi-tech Corporation, Tokyo, Japan).

### Organ indexes

The abdominal adipose and liver tissues of the hens were weighed in situ, and the organ indexes were measured as the following: organ indexes (g/kg) = organ weight/live body weight.

### Liver and jejunal morphology analysis

As previously described [22], liver and intestinal segments fixed in paraformaldehyde were embedded in paraffin wax, cut into slices, morphologically analyzed by hematoxylin and eosin (H&E) staining, and then liver paraffin sections were stained with Oil red O. The jejunum immobilized in a 2.5% glutaraldehyde solution was prepared specifically for the purpose of transmission electron microscope (TEM). Ultrathin slices were dehydrated in a graded ethanol series. Micrographs were obtained using the TEM as we previously described [22].

### TUNEL assay

The TUNEL assay was performed using the apoptosis detection kit (C1088, Beyotime Biotechnology, China). In summary, liver sections were first deparaffinized and rehydrated. Subsequently, they were incubated with proteinase K at 37 °C for 20 min to extract antigens. Following this, the sections were incubated with a mixture. The sections were incubated with a secondary antibody and subsequently counterstained.

### Cecal microbiota analysis

A TIANamp fecal DNA kit was used to extract genomic DNA from the cecal contents according to the manufacturer’s protocol [23]. The V3–V4 region of the 16S rDNA gene of the microbiota was amplified using the 338F/806R primer, and high-throughput sequencing was performed using the Illumina MiSeq PE300 platform of Shanghai Myllo Biotechnology Co., Ltd. High-throughput sequencing data were analyzed and visualized using the Majorbio Cloud Platform (www.majorbio.com).

### Cecal metabolome profiling

Based on the bacterial microbiota and phenotype results, the M2 (400 mg/kg MAG) and HFLP-induced CON groups were selected for non-target metabolome detection of the cecal contents. The LC–MS/MS analysis was performed using an ultra-performance liquid chromatography-tandem Fourier transform mass spectrometry (UHPLC-Q Exactive HF-X) system. Data-dependent acquisition (DDA) was used for MS/MS mass spectrometry acquisition, and the database data matrix after matching was uploaded to the free online Majorbio Cloud Platform (https://cloud.majorbio.com) for data analysis.

### RNA extraction and quantitative real-time PCR (qRT-PCR) analysis

RNA was extracted from the liver and jejunum tissue homogenates using FreeZol Reagent (R711-01, Vazyme) according to the manufacturer’s protocol. For reverse transcription, single-stranded cDNA was synthesized using a HiScript III 1st Strand cDNA Synthesis Kit (R312-01, Vazyme). The transcriptional changes of target genes were measured by qRT-PCR using the Taq Pro Universal SYBR qPCR Master Mix (Q712-02, Vazyme) and Bio-Rad CFX Connect™ real-time quantitative PCR system (Bio-Rad, Hercules, CA, USA) [24]. Briefly, each 20 μL reaction mixture contained 10 μL Master Mix, 1 μL template cDNA, 0.4 μL (10 μmol/L) of each primer, and 8.2 μL ddH_2_O. The qRT-PCR conditions were pre-denaturation at 95 °C for 30 s, followed by 40 amplification cycles at 95 °C for 5 s, 60 °C for 30 s, and 72 °C for 30 s. Table S1 lists the primer sequences used in this study. Target gene expression levels were standardized relative to the mRNA of the housekeeping gene (*β-actin*) mRNA according to the 2^−ΔΔCt^ calculation method [25].

### Hepatic inflammatory biomarkers analysis

The hepatic inflammatory response represented by the levels of inflammatory cytokines in the supernatant of liver homogenates, including interleukin-10 (IL-10), interleukin-6 (IL-6), tumor necrosis factor-α (TNF-α), and nuclear factor kappa B (NF-κB). The levels of these inflammatory cytokines were tested by using the corresponding ELISA kit specific for chickens (Wuhan ABclonal Technology Co., Ltd., China). The results of inflammatory cytokine levels were normalized against the total protein content for comparison between samples.

### Fecal microbiota transplantation (FMT)

We followed previously established protocols to prepare fecal bacterial suspensions [26–28]. Immediately after defecation, fresh fecal samples from hens belonging to the MAG and CON groups were promptly collected and then transported on ice to the laboratory to prepare a fecal bacterial suspension. The fecal slurry was filtered through stainless steel sieves of varying pore sizes to effectively eliminate any undigested particles. The fecal suspension liquid was then centrifuged (5804R; Eppendorf, Hamburg, Germany) to separate the supernatant from the precipitate. The precipitate was resuspended in sterile saline. The refrigerated fecal suspension was dissolved gently in the 37 °C water bath before FMT. After thawing, the fecal suspension was diluted in sterile saline to a standardized concentration of 1 × 10^9^ colony-forming units (CFU)/mL. Twenty FLHS laying hens were randomized into FMT-CON and FMT-MAG groups (10 hens in each group) and received an antibiotic cocktail (ampicillin, 10 mg/L; metronidazole, 100 mg/L; vancomycin, 50 mg/L; neomycin, 100 mg/L; and amphotericin B, 1 mg/L) for 1 week. Then hens in each group were administered 5 mL of the corresponding fecal bacteria suspension by gavage once daily for 30 d.

### Statistical analysis

Data were analyzed by using one-way ANOVA procedure of IBM SPSS Statistics 26.0 software (SPSS Inc., Chicago, IL, USA) followed by Tukey’s post-hoc tests. Statistical significance was set at *P* < 0.05. Values were expressed as mean ± standard deviation (SD). Image processing was performed in GraphPad Prism 9.5 (GraphPad Software Inc., San Diego, CA, USA).

## Results

### MAG improved laying performance in FLHS hens

The results (Table 2) showed that MAG improved laying performance evidenced by the increased LR and ADEM in the M1 and M2 groups as compared with the CON group after 9 weeks of MAG treatment (*P* < 0.05). In addition, hens in M2 group showed the highest LR and ADEM among groups (*P* < 0.05).

**Table 2:** Effect of MAG on the growth performance of laying hens (*n* = 9)

Items	CON	M1	M2	M3	*P*-value		
					**ANOVA**	**Linear**	**Quadratic**
LR, %	81.77 ± 0.048^b^	86.51 ± 0.033^a^	88.59 ± 0.017^a^	86.07 ± 0.011^ab^	0.009	0.029	0.003
ADEM, g/hen/d	47.03 ± 2.189^b^	49.89 ± 0.885^a^	51.02 ± 2.648^a^	49.07 ± 1.214^ab^	0.011	0.084	0.004
ADFI, g/hen/d	111.53 ± 4.37	113.28 ± 8.15	114.26 ± 3.47	113.23 ± 6.17	0.876	0.557	0.708
FCR	2.33 ± 0.082	2.25 ± 0.176	2.23 ± 0.086	2.31 ± 0.123	0.506	0.683	0.309

*LR* Laying rate, *ADEM* Average daily egg mass, *ADFI* Average daily feed intake, *FCR* Feed conversion ratio

^a,b^Values within a row with different letters differ significantly (*P* < 0.05)

### MAG mitigated hepatic lipid accumulation and liver damage in FLHS hens

**Fig. 1:**
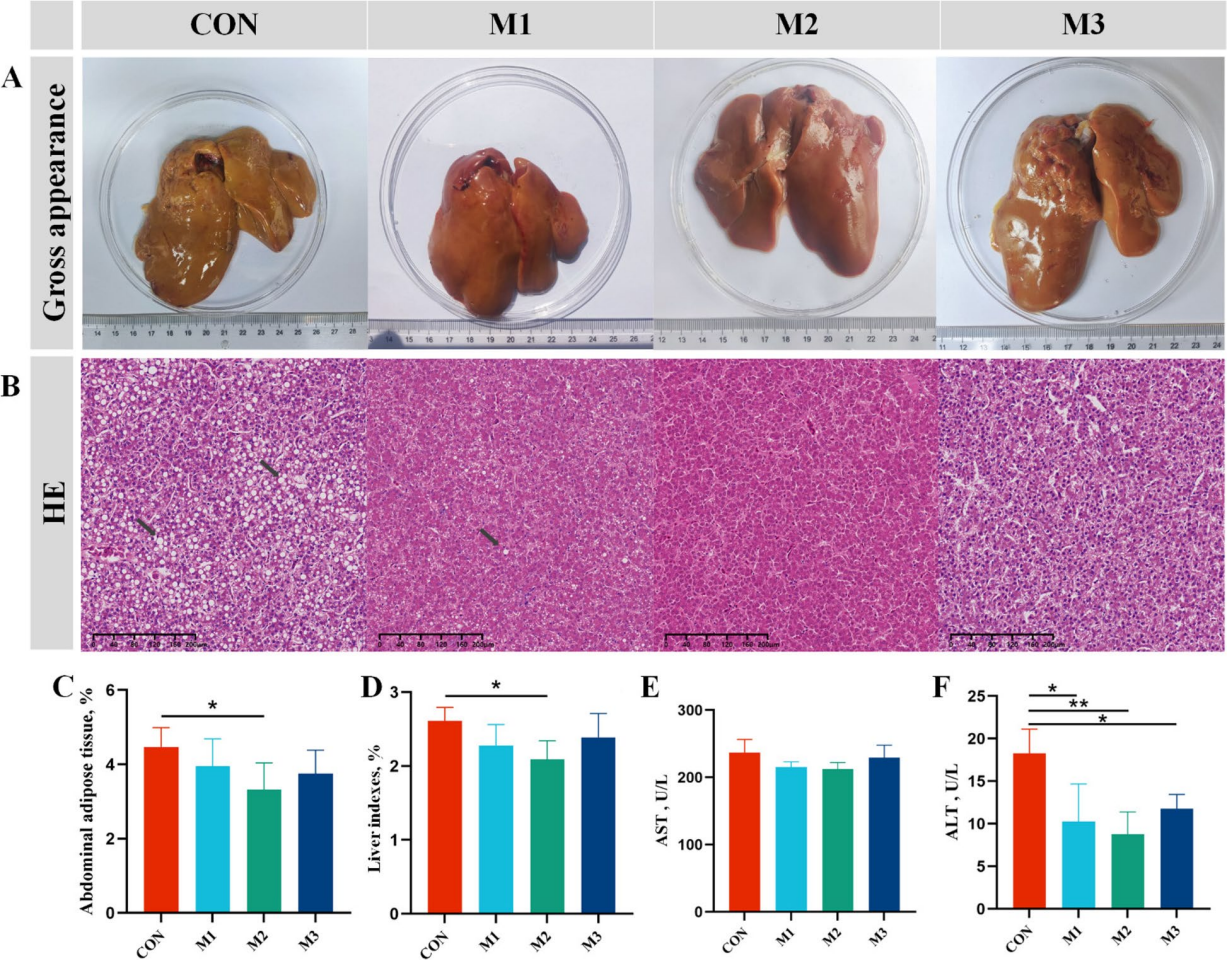
Magnolol relieved the structural damages in liver of FLHS hens. **A** Gross appearance of liver morphology. **B** The representative image of H&E staining of liver morphology. **C** and **D** Weight of abdominal adipose and liver tissue as a percentage of body weight. **E** and **F** Serum biochemical indexes of laying hens. Abbreviations: FLHS: fatty liver hemorrhagic syndrome; AST, aspartate transferase; ALT, alanine transaminase. Data were showed as mean ± SD. *n* = 6 hens per group. ^*^P < 0.05, ^**^P < 0.01

**Fig. 2:**
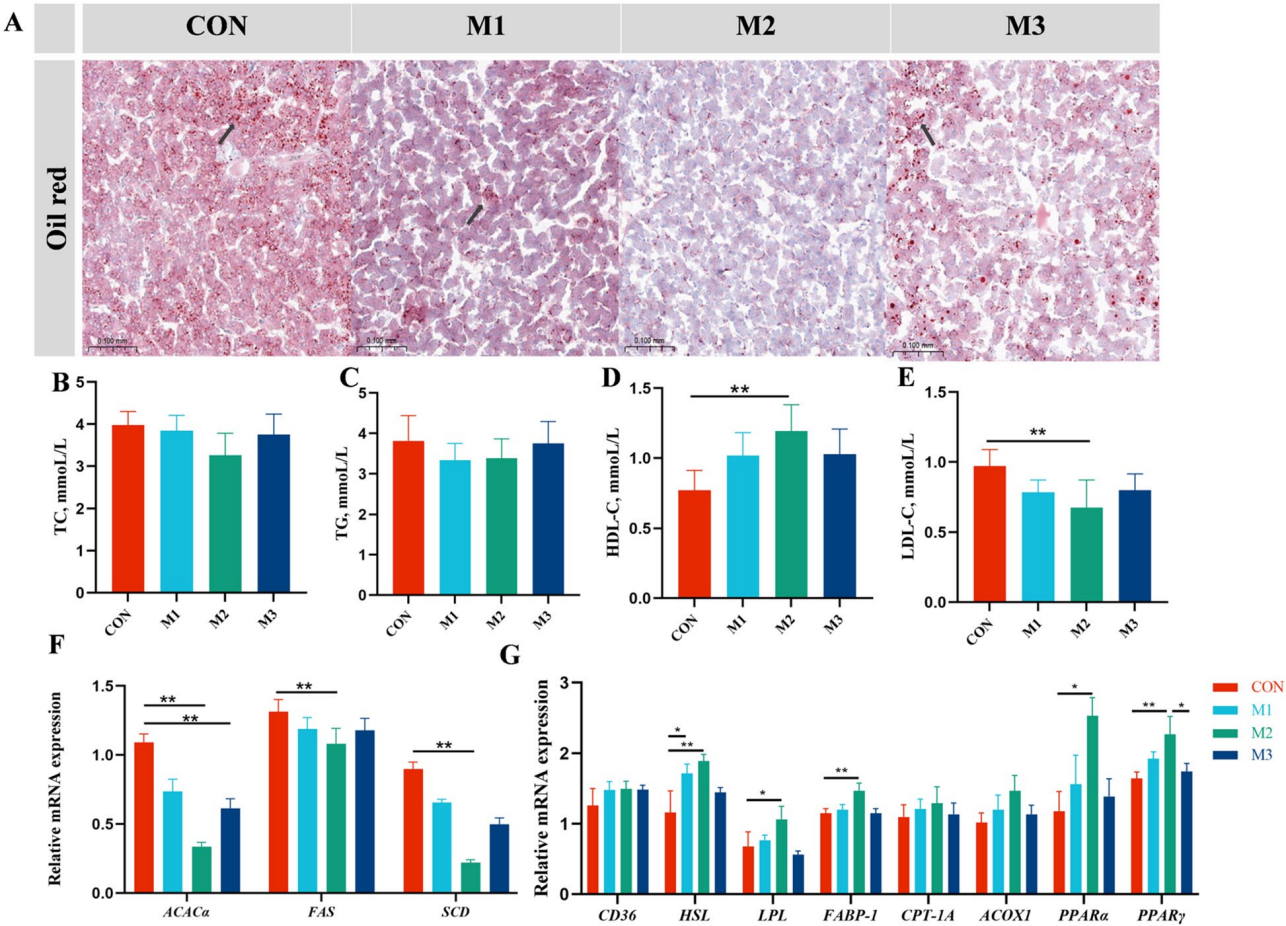
Magnolol relieved the lipid metabolism disorder in liver of FLHS hens. **A** The representative image of Oil red O staining of liver. **B**–**E** Serum biochemical indexes of laying hens. **F** Relative mRNA expression levels of lipid synthesis related genes. **G** Relative mRNA expression of lipid transport and lipolysis related genes. Abbreviations: TC, total cholesterol; TG, triglyceride; HDL-C, high-density lipoprotein cholesterol; LDL-C, low-density lipoprotein cholesterol; *ACACα*, acetyl-CoA carboxylase alpha; *FAS*, fatty acid synthase; *SCD*, stearoyl-CoA desaturase; *CD36*, fatty acid translocase; *HSL*, hormone-sensitive lipase; *LPL*, lipoprotein lipase; *ACOX1*, acyl-CoA oxidase 1; *FABP-1*, fatty acid binding protein 1; *CPT-1A*, carnitine palmitoyl transferase 1A; *PPARα*, peroxisome proliferator activated receptor alpha; *PPARγ*, peroxisome proliferator activated receptor gamma. Data were showed as mean ± SD. *n* = 6 hens per group. ^*^P < 0.05, ^**^P < 0.01

To evaluate the mitigating effect of MAG on hepatic lipid accumulation and injury in FLHS hens, we analyzed the histomorphology of liver tissues and serum concentrations of hepatic lipid metabolism (TG, TC, HDL-C, and LDL-C) and hepatic damage biomarkers (AST and ALT). The sizes of the liver organs and the abdominal and liver indexes were significantly decreased in M2 group compared to those in the CON group (Fig. 1A, C and D). H&E and Oil red O results showed that the fat vacuoles were obviously observed in the liver tissue of the CON hens, and these symptoms were relieved in MAG-treated groups (Fig. 1B and 2A). ALT activity and LDL-C levels significantly decreased in serum of hens from M2 group (Fig. 1F and 2E, *P* < 0.01).

To elucidate the fundamental molecular mechanism underlying MAG’s enhancement of lipid metabolism, we assayed the mRNA expression profiles of crucial genes involved in lipogenesis, lipolysis, fatty acid oxidation, and lipid translocation. As shown in Fig. 2F and G, the mRNA expression levels of lipogenesis-related genes (*ACACα*,* FAS* and *SCD*) were significantly downregulated in the liver of hens in M2 group compared to those in the CON group (*P* < 0.01). Additionally, the mRNA expression of genes involved in lipolysis and fatty acid oxidation (*HSL*,* LPL*,* FABP-1, PPARα*, and *PPARγ*) were significantly upregulated in the liver of hens in M2 group compared to the CON group (*P* < 0.05). However, there were no significant differences in the mRNA expression levels of *CD36*, *ACOX1*, or *CPT-1A* among groups (*P* > 0.05).

### MAG inhibited apoptosis and immunoreaction in FLHS hens

**Fig. 3:**
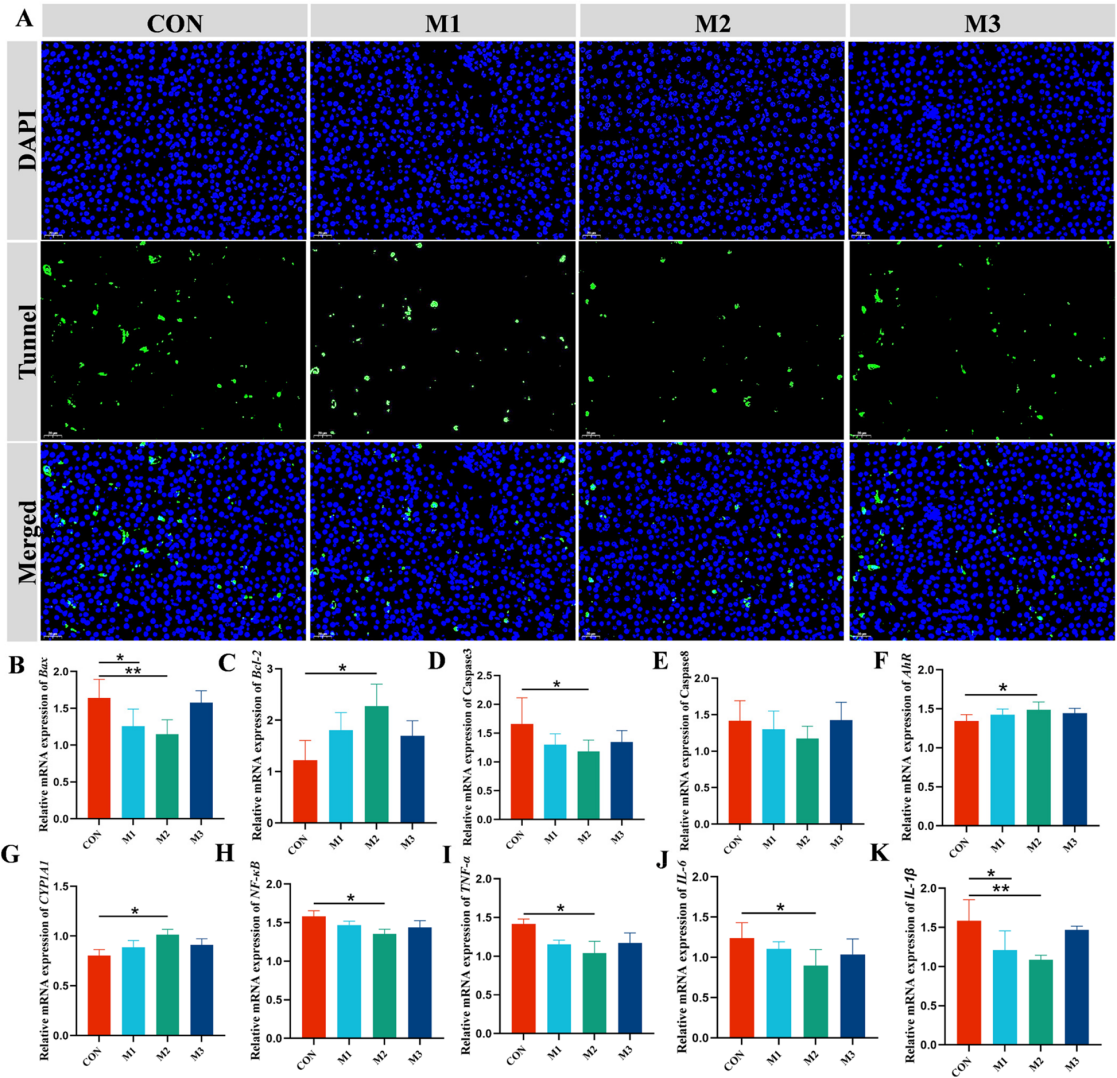
Magnolol attenuated the apoptosis and inflammatory response in liver of FLHS hens. **A** Representative TUNEL (green) immunofluorescence staining images of liver tissue. **B**–**E** The mRNA expression levels of apoptosis-related genes. **F**–**K** The relative mRNA expression of genes related with inflammatory response. Abbreviations: *Bax*, Bcl2-associated X; *Bcl-2*, B-cell lymphoma-2; *AhR*, aryl hydrocarbon receptor; *CYP1A1*, cytochrome P450 family 1 subfamily A member 1; *NF-κB*, nuclear factor kappa B; *TNF-α*, TNF-alpha; *IL-6*, interleukin 6; *IL-1β*, interleukin 1 beta. Data were showed as mean ± SD. *n* = 6 hens per group. ^*^P < 0.05, ^**^P < 0.01

TUNEL staining showed that the intensity of green fluorescence was significantly reduced after MAG supplementation as compared to the CON group (Fig. 3A). Accordingly, the M2 group downregulated the gene expression of Bcl2-associated X (*Bax*) and Caspase3, and significantly upregulated the B-cell lymphoma-2 (*Bcl-2*) mRNA expression level in liver tissue (Fig. 3B–E). The gene expression levels of aryl hydrocarbon receptor (*AhR*) and cytochrome P450 family 1 subfamily A member (*CYP1A1*) were significantly upregulated in liver of hens from M2 group. The mRNA expression levels of genes related to the hepatic inflammatory response are shown in Fig. 3F–K. The M2 group markedly downregulated the pro-inflammatory cytokines (*NF-κB*, *TNF-α*, *IL-6*, and *IL-1β*) mRNA expression levels in comparison to the CON group.

### MAG improved the intestinal morphology and barrier function

**Fig. 4:**
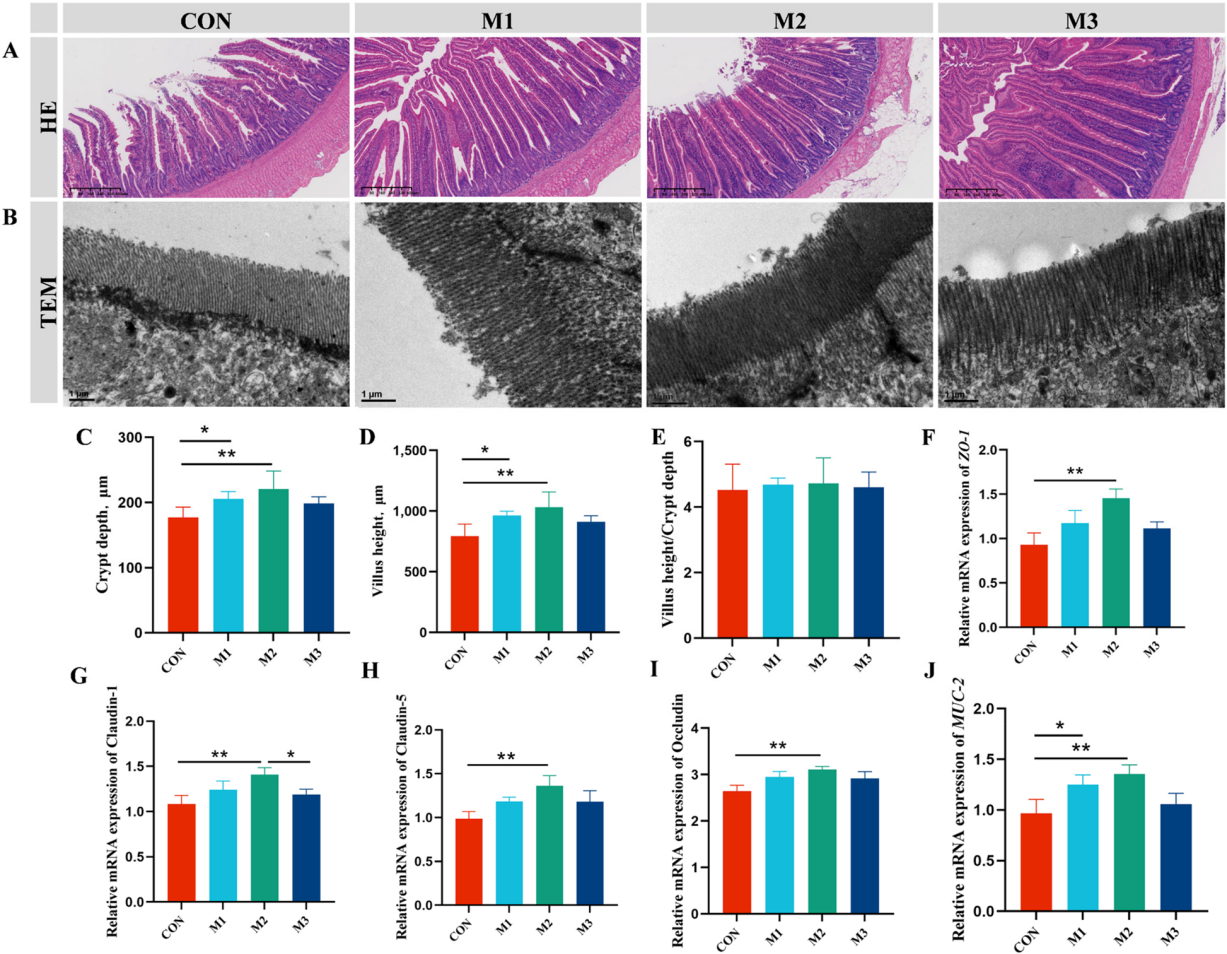
Magnolol improved the jejunal morphology and barrier functions of FLHS hens. **A** The representative H&E staining images of jejunal morphologies. **B** Transmission electron micrographs of the jejunal microvilli. **C**–**E** Villus height, crypt depth, and villus height to-crypt depth ratio. **F**–**J** Relative mRNA expression of tight junction related genes. Abbreviations: *ZO-1*, Zonula occludens-1; *MUC-2,* Mucin 2. Data were showed as mean ± SD. *n* = 6 hens per group. ^*^P < 0.05, ^**^P < 0.01

The histological morphology depicted in Fig. 4A revealed a normal appearance in the M2 group, characterized by regular, intact, and closely packed intestinal villi, crypts, and intact mucosal structures, and villus height and crypt depth (Fig. 4C and D) were significantly increased in M2 group (*P* < 0.01), whereas no significant effects (*P* > 0.05) were observed in the ratio of villus height to crypt depth (Fig. 4E). TEM analysis revealed that hens receiving MAG exhibited a higher density of microvilli and elongated tight junctions in the jejunum than those in the CON group (Fig. 4B). The gene expression of tight junctions corroborated these findings that MAG upregulated the mRNA expression of zonula occludens-1 (*ZO-1*), Claudin-1, Claudin-5, Occludin, and Mucin-2 (*MUC-2*) in the jejunum at varying degrees (Fig. 4F–G). Furthermore, supplementation with 400 mg/kg of MAG (M2) considerably increased the mRNA expression of the aforementioned genes (*P* < 0.05).

### MAG changed the diversity and composition of intestinal microbiota

**Fig. 5:**
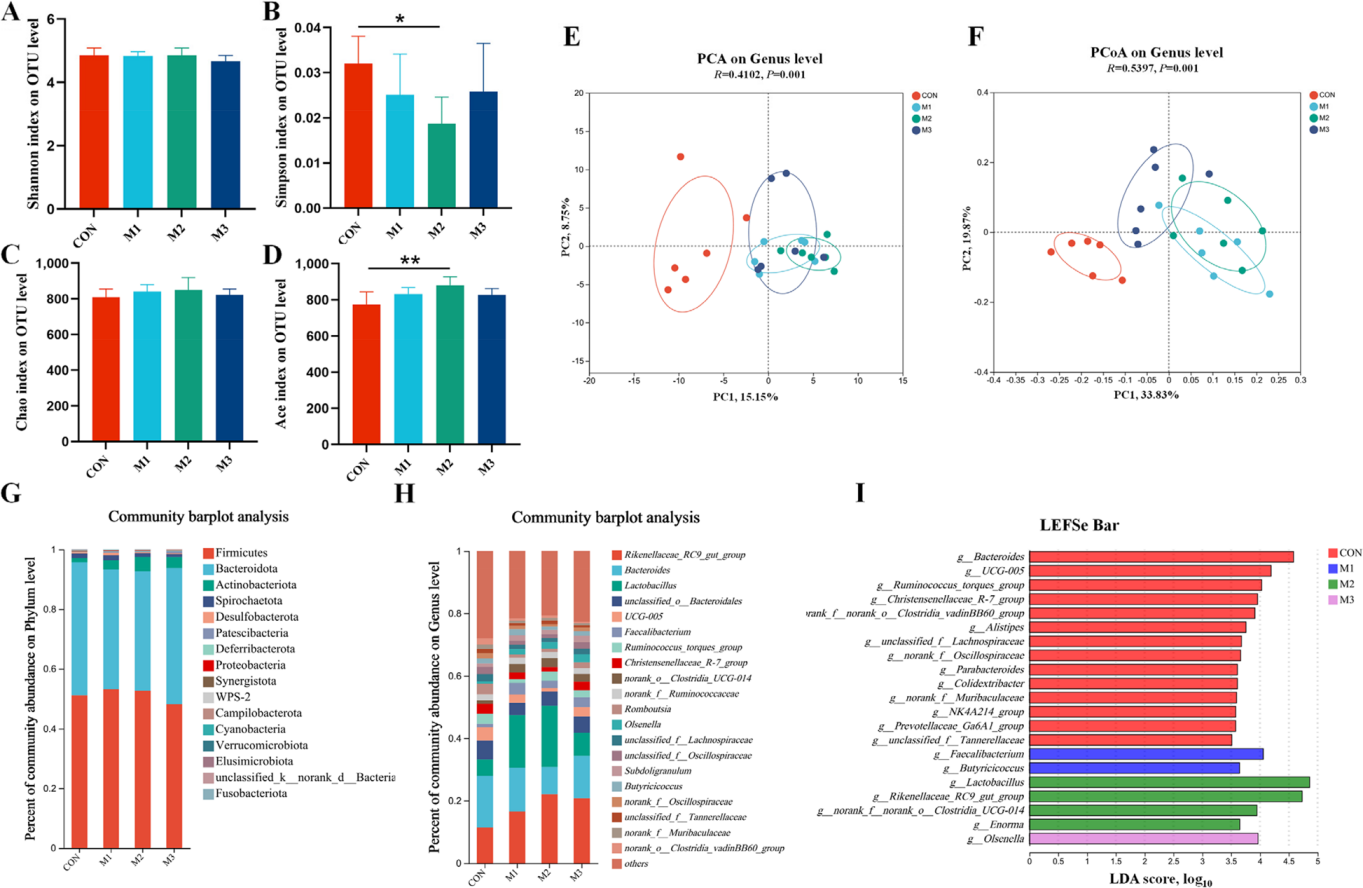
Magnolol changed the diversity and composition of intestinal microbiota of FLHS hens. Panels (**A–D**) were the shannon index, chao index, simpson index and ace species of cecal microflora; Panel (**E** and **F**) were the principal component analysis (PCA) and principal coordinate analysis (PCoA) plot of the cecal microflora composition at genus level. **G** and **H** Percent of community abundance of the cecal microflora community at the phylum and genus level. **I** LDA effect size (LEfSe) analyzed (LDA score 3.5) from the phylum level to the genus level. Data were showed as mean ± SD. *n* = 6 hens per group. ^*^P < 0.05, ^**^P < 0.01

Alpha diversity encompassed both diversity (Shannon and Simpson index) and richness (Chao1 and Ace index) of the gut microbiota. Hens fed 400 mg/kg of MAG exhibited a notable decrease in the Simpson index (*P* < 0.05; Fig. 5B) and a significant increase in the Ace index (*P* < 0.01; Fig. 5D), suggesting that an enhancement in bacterial richness and diversity upon MAG treatment. Principal component analysis (PCA) and principal coordinate analysis (PCoA) were utilized using Bray-Curti’s distance to visualize alterations in the microbiota community. At the genus level, samples from the MAG group clustered together and were distinct from the CON group, indicating a significant difference (*P* < 0.05) in the gut microbiota structure following MAG treatment (Fig. 5E and F). For the composition of intestinal microbiota, the relative abundances of the gut microbiota at the phylum and genus levels changed in hens treated with MAG. Firmicutes and Bacteroidota were the most abundant phyla in cecal microbiota (Fig. 5G). At genus level, *Rikenellaceae_RC9_gut_group*, *Bacteroides*, *Lactobacillus*, *Faecalibacterium*, *Romboutsia*, *Olsenella*, *Subdoligranulum*, and *Butyricicoccus* were the dominant bacteria (Fig. 5H).

**Fig. 6:**
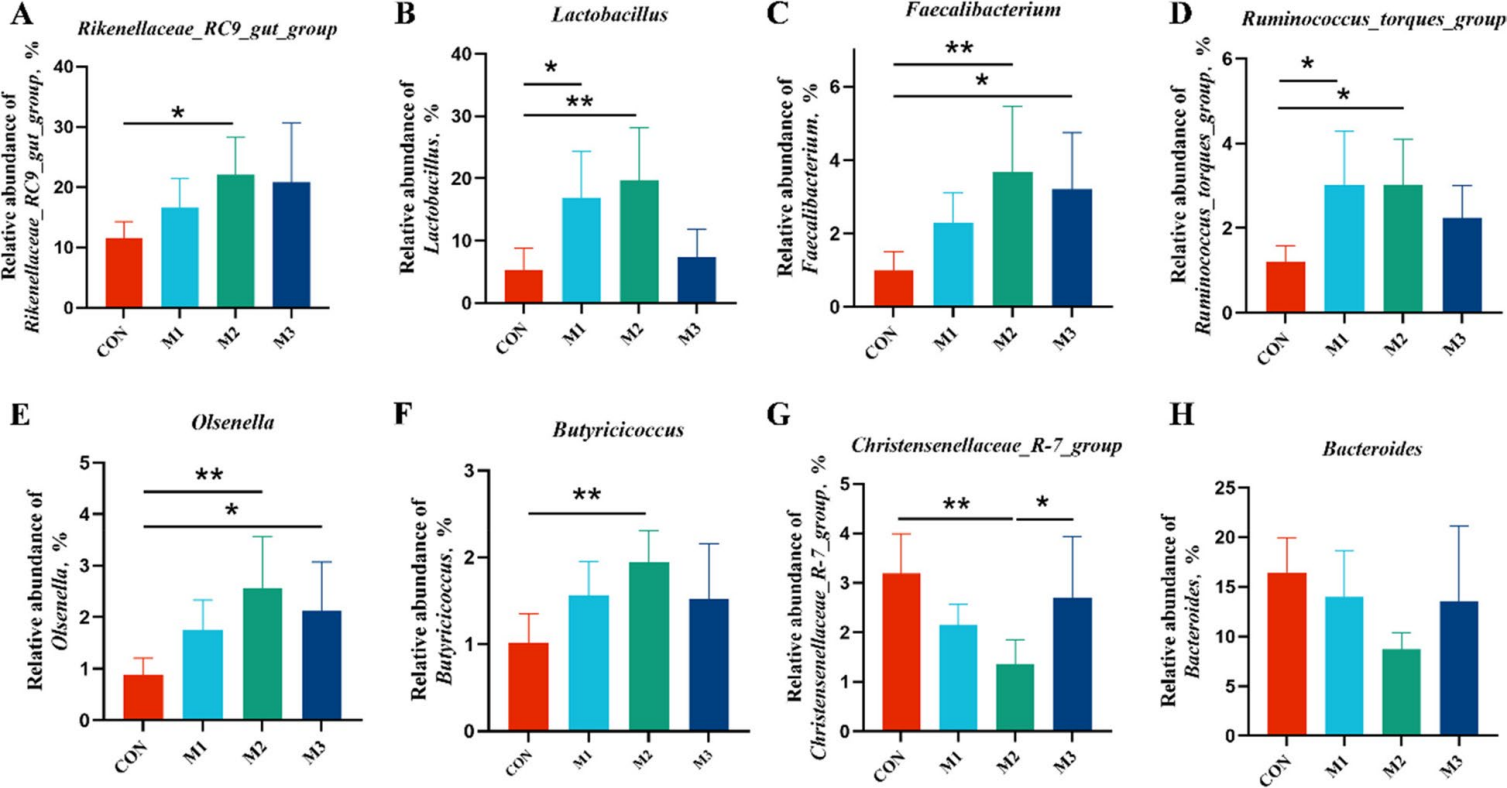
Effects of dietary magnolol supplementation on specific microbiota in cecal contents. **A**–**H** The relative abundance of cecal microbiota at the genus level with significant variations and specific functions. Data were showed as mean ± SD. *n* = 6 hens per group. ^*^P < 0.05, ^**^P < 0.01

To further analyze the gut microbiota involved in MAG treatment, significantly different genera between the MAG-treated and CON groups were identified using LEfSe analyses. As shown in Fig. 5I, 21 taxa biomarkers in the four groups were identified with an LDA score > 3.5, which belonged to the phyla Firmicutes, and Bacteroidota. The CON group exhibited higher abundances of *Bacteroides*, *UGG-005*, and *Christensenellaceae_R-7_group*. Meanwhile, the abundances of *Lactobacillus*, *Faecalibacterium*, *Butyricicoccus*, *Olsenella*, and *Rikenellaceae_RC9_gut_group* were enriched in MAG-treated laying hens. The relative abundance of these specific microbiota in cecal contents were shown in Fig. 6, the abundance of *Rikenellaceae_RC9_gut_group*, *Lactobacillus*, *Faecalibacterium*, *Ruminococcus_torques_group*, *Olsenella*, and *Butyricicoccus* were remarkably increased in the M2 group (*P* < 0.05), whereas the abundance of *Christensenellaceae_R-7_group* was markedly decreased (*P* < 0.05*,* Fig. 6 A–H) in comparison with the CON group.

### MAG altered the cecal tryptophan metabolome profiling

**Fig. 7:**
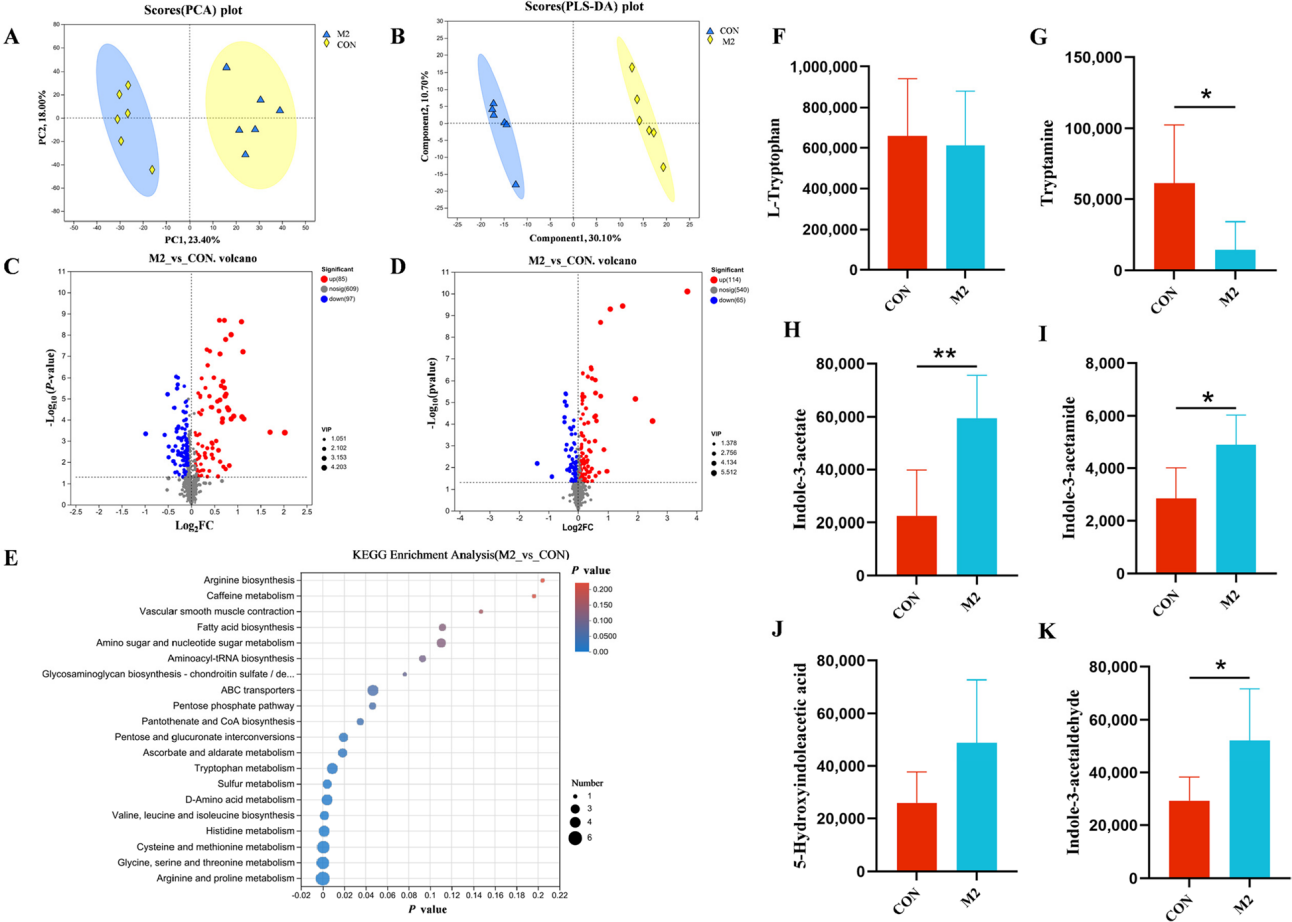
Magnolol altered tryptophan metabolism in cecal contents of FLHS hens. **A **and** B** Principal component analysis (PCA) plot and PLS-DA scores in positive ion modes. **C** and **D** Volcano plot showing the differential variables between CON group and M2 group in negative and positive ion modes. **E** Pathway enrichment of differentially expressed metabolites was analyzed using the pathway enrichment statistical scatterplot. **F**–**K** Tryptophan and its metabolite contents in different treatments. Data was showed as mean ± SD. *n* = 6 hens per group. ^*^P < 0.05, ^**^P < 0.01

To elucidate the metabolic regulation mechanism of MAG in mitigating lipid dysregulation in FLHS hens, a comparative cecal metabolome analysis was conducted between hens in CON and M2 groups. PCA of cecal samples revealed metabolomic differences associated with dietary MAG (Fig. 7A). In order to confirm this connection, partial least squares-discriminant analysis (PLS-DA) of the cecum samples showed metabolomic differences between the two groups, suggesting that the metabolites in the CON and M2 groups were classified into two categories (Fig. 7B). According to the volcano plots, the M2 group had 85 upregulated and 97 downregulated metabolites compared to the CON group in negative ion mode (Fig. 7C). Similarly, 62 upregulated and 75 downregulated metabolites were identified in the positive ion mode (Fig. 7D). Differential metabolites were enriched according to the KEGG pathway analysis. These metabolites were primarily enriched in arginine and proline metabolism, as well as glycine, serine, threonine, and tryptophan metabolism. Tryptophan metabolism was a highly significant enrichment term in the KEGG analysis (Fig. 7E). Tryptamine (TRM), a metabolite in the tryptophan metabolic pathway, was significantly decreased, and indole-3-acctate (IAA), indole-3-acctamide (IAM), and indole-3-acctaldehyde (IAAld) were significantly increased in M2 group as compared with the CON group (Fig. 7G–I and K), whereas the changes in L-tryptophan (TRP) and 5-hydroxyindoleacctic (5-HAA) were not significant (Fig. 7F and J).

### Correlation analysis between indicators of metabolic disorders in FLHS and cecal microbiota

**Fig. 8:**
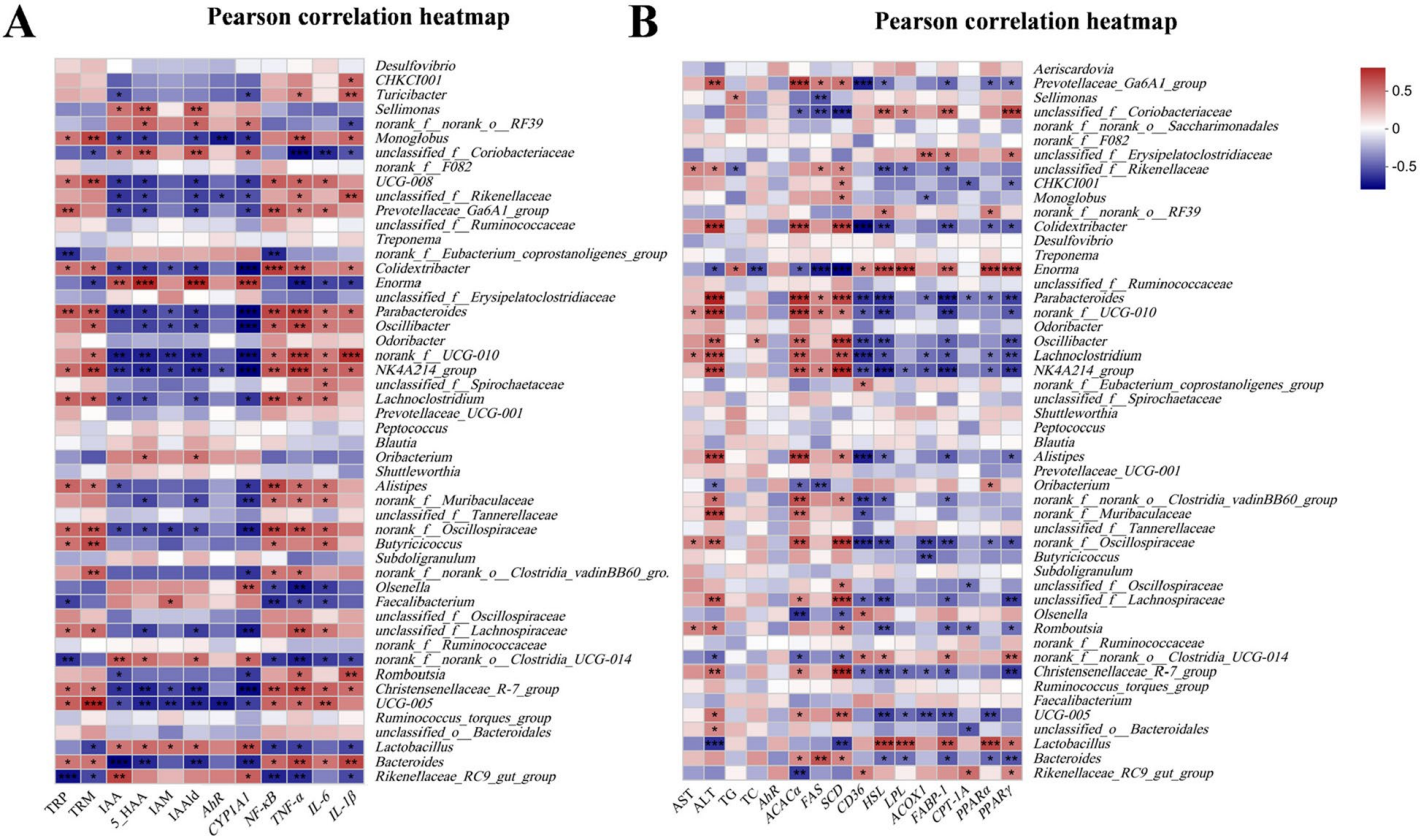
Pearson correlation between tryptophan metabolites, inflammatory response, liver injury biomarkers, as well as lipid metabolism-related indicators and the differentially abundant intestinal microbiota at the genus level (top 50 genera). **A** Pearson correlation analysis between intestinal microbiota and tryptophan metabolites as well as inflammatory cytokines. **B** Pearson correlation between intestinal microbiota and the markers of liver injury and lipid metabolism. The intensity of the colors represented the degree of association (red, negative correlation; blue, positive correlation). Abbreviations: TRP, L-Tryptophan; TRM, Tryptamine; IAA, Indole-3-acctate; 5-HAA, 5*-*Hydroxyindoleacetic acid; IAM, Indole-3-acctamide; IAAld, Indole-3-acctaldehyde. Significant correlations were marked by ^*^P < 0.05, ^**^P < 0.01 and ^***^P < 0.001

Pearson’s correlation analyses were conducted between the tryptophan metabolites, pro-inflammatory factors, liver injury biomarkers, as well as lipid metabolism-related indicators and the differentially abundant intestinal microbiota at the genus level (top 50 genera). The correlation between tryptophan metabolites, pro-inflammatory factors, and the differentially abundant microbiota is shown in Fig. 8A. The metabolic substrates TRP and TRM were negatively correlated with the beneficial bacteria *Rikenellaceae_RC9_gut_group*, *Faecalibacterium*, and *Lactobacillus*. The IAA, 5-HAA, IAM and IAAld levels were positively correlated with *Lactobacillus* while the pro-inflammatory factor (*NF-κB*, *TNF-α* and *IL-1β*) was strongly negatively associated with beneficial bacteria (e.g., *Lactobacillus* and *Faecalibacterium*) and positively associated with harmful bacteria (e.g., *Bacteroides* and *Alistipes*). As shown in Fig. 8B, the abundance of *Christensenellaceae_R-7_group*, *Alistipes*, *Oscillibacter*, and *Romboutsia* were positively correlated with ALT activity, while the levels of *Lactobacillus* were negatively associated with ALT activity.

### FMT attenuated the liver damages in FLHS hens

**Fig. 9:**
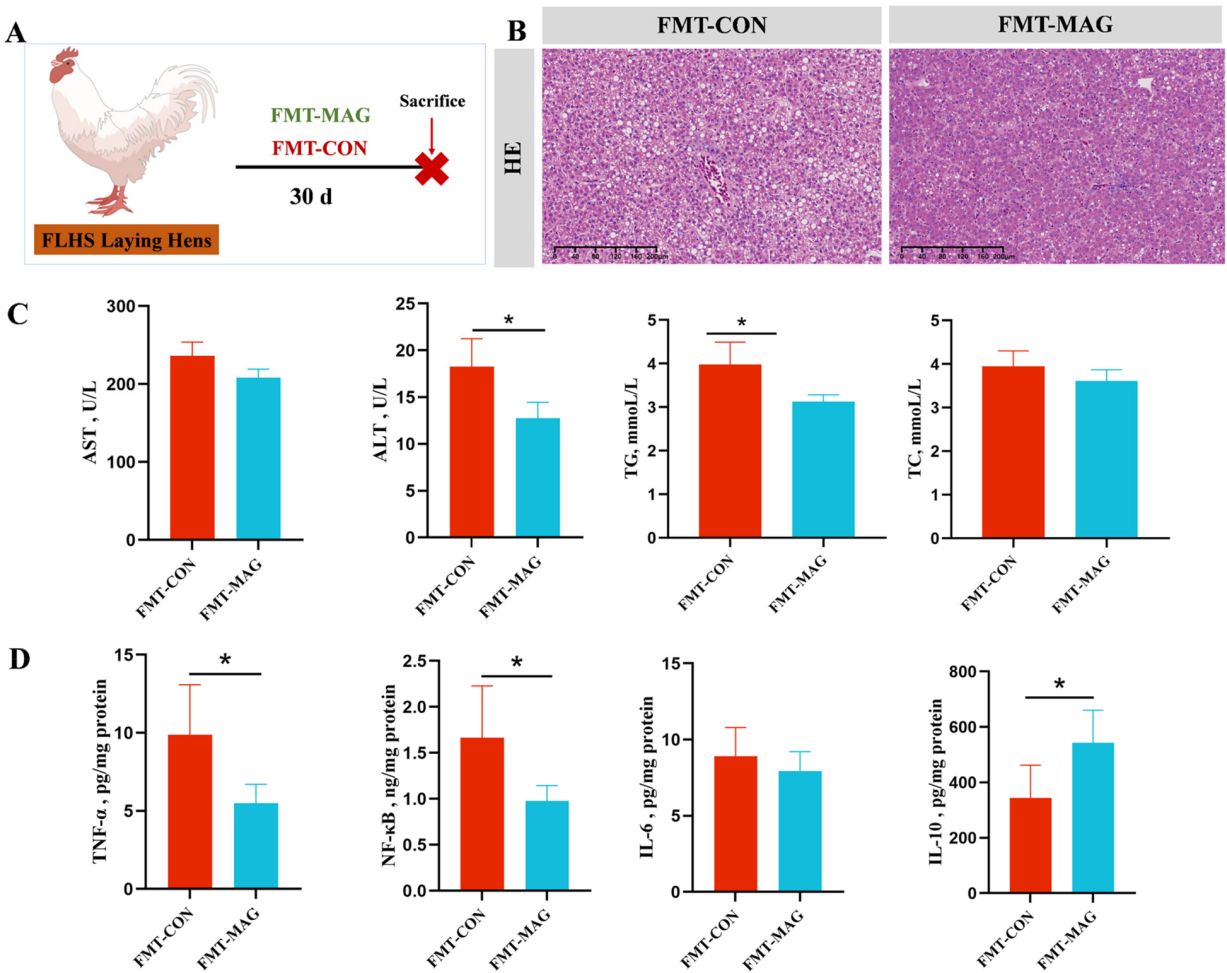
FMT attenuated HFD-induced FLHS in laying hens. **A** Schematic diagram of the experimental procedure. **B** Representative photomicrographs of liver tissues with H&E staining. **C** Serum biochemical indexes of laying hens. **D** The inflammatory cytokine contents in liver tissue. Abbreviations: IL-10, Interleukin 10. Data were showed as mean ± SD. *n* = 10 hens per group. ^*^P < 0.05, ^**^P < 0.01

To assess the therapeutic potential of MAG-modulated gut microbiota in FLHS hens, a fecal microbiota transplantation (FMT) experiment was performed (Fig. 9A). H&E staining showed that several fat vacuoles were clearly observed in the liver tissue of the FMT-CON group, whereas the symptoms in the FMT-MAG group were relieved (Fig. 9B). Oral treatment with fecal bacterial suspensions from MAG-treated donors significantly reduced ALT activity and TG levels in serum of FLHS hens (Fig. 9C). ELISA results showed that FMT-MAG decreased the TNF-α and NF-κB levels and increased IL-10 levels in liver as compared with FMT-CON group (Fig. 9D).

## Discussion

Fatty liver hemorrhagic syndrome is one of the most serious hepatic metabolic disorders, leading to decreased egg production and unexpected mortality in laying hens, and thus causing significant losses to the farming industry. Although methods for regulating the FLHS in laying hens have long been explored, there is no effective method for solving this problem. In this study, MAG extracted from *Magnolia officinalis* were used to treat FLHS hens induced by HFLP diet to elucidate the regulatory mechanism of MAG in alleviating the liver injury. Previous studies have confirmed that production performance will be affected to varying degrees in aged or FLHS hens due to the hepatic lipid metabolism dysfunction as compared with the peak laying period [29–31]. In the present study, we found that the laying rate, average daily egg mass, and feed conversion ratio of FLHS hens were improved after dietary MAG supplementation.

The most prominent features of FLHS-related diseases are imbalanced lipid metabolism and hepatic lipid deposition, along with hepatic steatosis [32]. In the case of liver damage, the activity of ALT and AST in serum, the major transaminases involved in the synthesis of non-essential amino acids, will be elevated [33]. Some studies have shown that TG and TC levels significantly increase in FLHS hens [34, 35]. Accumulation of TG in the liver is generally considered to be the main feature of liver injury [36]. HDL-C and LDL-C are critical for lipid transport, and their densities are related to cholesterol transport. HDL-C is thought to have a protective role in hepatic lipid metabolism, whereas LDL-C is harmful to the liver [37]. The intraperitoneal administration of 0.01 μg/kg of MAG effectively attenuated hepatic lipid peroxidation and demonstrated efficacy in restoring the aberrant biochemical parameters and pathological manifestations associated with acetaminophen-induced acute liver injury [38], which is in line with our findings that MAG decreased the liver-associated damage and lipid metabolic disorder parameters and improved the symptoms of FLHS hens.

FAS*,* ACACα*,* and SCD play crucial roles in de novo synthesis of lipids. FAS is a pivotal enzyme facilitating the synthesis of fatty acids. ACACα serves as a crucial rate-limiting enzyme in fatty acid synthesis, while SCD functions as the primary catalyst in the process of lipogenesis [39]. In this study, the mRNA expression of *ACACα*, *FAS*, and *SCD* genes in liver was significantly down-regulated to inhibit lipid synthesis, while the expression of genes related to fat metabolism and transport was significantly up-regulated, thereby reducing fat accumulation in FLHS hens. As a result, the lipid deposition decreased after dietary supplementation with MAG as compared with the FLHS hens.

During aging, gut barrier damage and gut microbiota disorders can activate inflammatory responses or induce immune activation [40–42]. When the intestinal barrier is damaged, intestinal permeability increases, leading to the transfer of multiple pathogen-associated molecular patterns into the bloodstream, and eventually induce inflammatory responses in invade tissues and organs [43, 44]. Therefore, intestinal barrier dysfunction and microbiota dysregulation have emerged as promising candidates for the treatment of various diseases, particularly liver diseases [45]. Damage to the intestinal mucosal barrier is a common phenomenon in functional digestive diseases or inflammatory bowel diseases, and the reduced expression of tight junctions in the mucosa and breakdown of tight junctions may even lead to structural dysregulation of the intestinal microbiota; however, it can be alleviated by MAG [46, 47]. In line with the above studies, the jejunum tissue of FLHS hens showed intact intestinal structure and villi, significantly increased villus height and crypt depth, and upregulated expression of tight junctions related genes after supplementation with MAG, indicating that the decreased intestinal barrier function in FLHS hens could be improved by MAG supplementation to a certain extent.

HFLP-induced dysbiosis of intestinal microbiota may disrupt host energy metabolism, and contribute to the development of FLHS [48]. The addition of MAG reduced the relative abundance of thick-walled bacterial phyla in the cecum, which may help mitigate hepatic lipid deposition. Nonalcoholic fatty liver disease is characterized by more Firmicutes, fewer Bacteroidota, and a decreased Firmicutes/Bacteroidota [49]. It has been confirmed that Bacteroidota promote the development and deterioration of inflammatory bowel disease by producing enterotoxins, degrading mucin, activating TLR, and stimulating the secretion of pro-inflammatory cytokines [50, 51]. *Rikenellaceae_RC9_gut_group* has shown beneficial effects in both human and animal populations [52], and may be a regulator of fat [53]. The significance of the elevated levels of *Rikenellaceae_RC9_gut_group* lies in its potential to prevent cardiovascular and metabolic diseases associated with visceral fat [54]. *Lactobacillus* have the ability to improve lipid distribution, restore gut microbiota diversity, and are key members of the beneficial gut microbiota [55]. In rats fed an HFD, *Lactobacillus* reduced serum LDL-C and TC levels. It was found that the abundance of *Lactobacillus* was positively correlated with the intestinal permeability of rats [56]. Specifically, *Lactobacillus* has a significant effect on the regulation of host metabolism, particularly in maintaining the integrity of intestinal barrier function through the production of tryptophan metabolites [57]. In this study, *Lactobacillus* were positively correlated with IAA, IAM, and IAAld, and negatively correlated with TRM. In addition, *Rikenellaceae_RC9_gut_group* has also shown consistent correlations.

The gut microbiota efficiently metabolizes tryptophan to indole [57], which can regulate lipid metabolism [58, 59]. Indole tryptophan metabolites produced by gut microbiota metabolism are endogenous ligands for the aryl hydrocarbon receptor *AhR* [60]. It has been demonstrated that the gut microbiota-dependent metabolite indole-3-acetate can directly modulate the inflammatory response in hepatocytes. Indole-3-acetate acts on hepatocytes to attenuate cytokine-mediated upregulation of adipogenesis, and these effects of indole-3-acetate on hepatocytes are *AhR*-dependent [61]. Studies have demonstrated that the upregulation of *AhR* expression in the livers of mice administered *Lactiplantibacillus plantarum* P101 can effectively alleviate alcoholic liver injury, which is potentially attributed to intestinal microbiota-mediated IAM [62]. These findings provide detailed confirmation that intestinal microbiota and their tryptophan metabolites play pivotal roles in host metabolism. To further corroborate this, the intestinal microbiota modified by MAG was transplanted into FLHS hen. Oral treatment with fecal bacterial suspensions from MAG-treated donors attenuated the liver damages in FLHS hens, revealing that altered microbiota and metabolites alleviated liver steatosis and inflammation.

HFLP diet intake has been implicated in inducing lipid peroxidation in the liver, as established in the nutritional and health literature [63, 64]. Oxidative stress is a crucial mechanism underlying liver-related diseases, particularly by inducing dysfunction in hepatic lipid metabolism [65]. Notably, oxidative stress can activate inflammatory cascades and facilitate apoptosis, thereby promoting the secretion of pro-inflammatory cytokines such as IL-1β, IL-6, and TNF-α [66, 67]. Decreased ratios of *Bcl-2* and *Bax* can trigger the release of cytochrome C from the mitochondria, ultimately culminating in cell death [68]. In this study, we observed upregulation of the anti-apoptotic factor *Bcl-2* and suppression of Caspase-3 and Caspase-8 activation in MAG-treated hens compared to the FLHS group. In addition to regulating apoptosis, MAG increased the expression of *AhR* and its target gene *CYP1A1*, suggesting that the regulation of liver inflammation may be mediated by *AhR* downstream gene regulation.

TNF-α plays a pivotal role in initiating and propagating inflammatory responses [69]. This cytokine not only stimulates lipid peroxidation but also activates oxidative stress response genes, thereby amplifying and prolonging the inflammatory process [70]. Additionally, oxygen free radicals released during inflammation can activate *NF-κB*, leading to an upregulation of *TNF-α* expression [71, 72]. Notably, TNF-α can trigger the release of IL-6 from Kupffer cells in the liver, impeding the transport and secretion of TG [73]. Furthermore, elevated levels of *TNF-α* can result in hepatic TG storage and steatosis, which triggers the activation of *NF-κB*, creating vicious cycles that exacerbates liver injury [74]. Our findings indicate that MAG can attenuate the activation of inflammatory pathways, specifically downregulated the gene expression of *TNF-α*, *IL-6*, and *NF-κB*, through the activation of *AhR* and its downstream gene. This mechanism mitigates the effects of the inflammatory cascade and subsequently diminishes liver damage.

## Conclusions

**Fig. 10:**
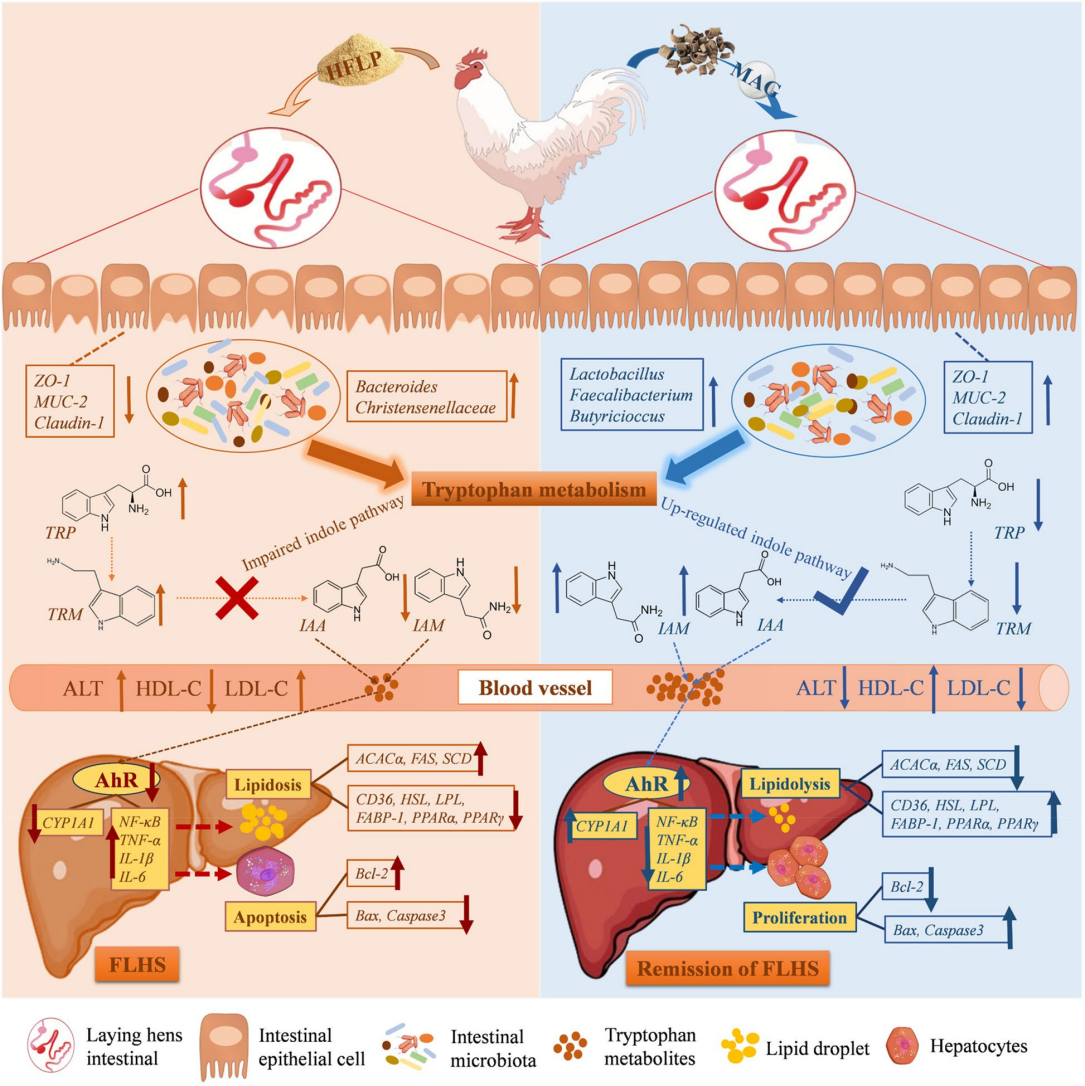
Schematic diagram of the regulatory network for the mitigating effect and mechanism of MAG on liver injury in FLHS laying hens. The arrows next to the names of microbes, genes, and metabolites indicate an increase (up) or decrease (down) in microbial abundance, gene expression, and metabolite content

The present study explained the mitigating effect and mechanism of MAG on liver injury in FLHS hens, based on a comprehensive multi-omics analysis. Laying hens with FLHS resulted in lower egg production performance, while dietary supplementation with 400 mg/kg of MAG significantly improved laying performance and attenuated liver injury and lipid metabolism disorder. These effects may be mediated by MAG-altered intestinal barrier function, remodeling of the gut microbial structure and composition, and subsequent effects on tryptophan metabolism. The products of tryptophan metabolism, such as IAA, IAM, and IAAld, can activate hepatic *AhR* and its target genes, thus alleviating hepatic inflammation and damage of FLHS hens (Fig. 10). Therefore, this study proposes a promising intervention using MAG as a functional feed additive, and provides a new approach for preventing liver diseases by targeting the gut microbiota and its metabolites.

## Supplementary Information

**Additional file 1: Table S1**. Sequences of real-time PCR primers.

## Data Availability

Datasets used or analyzed in this study are available by reasonable request from the corresponding author.

## Abbreviations

ACACα: Acetyl-CoA carboxylase
ACOX1: Acyl-CoA oxidase 1
ADFI: Average daily feed intake
ADEM: Average daily egg mass
AhR: Aryl hydrocarbon receptor
ALT: Alanine transferase
AST: Aspartate transferase
Bax: BCL2-associated X
Bcl-2: B-cell lymphoma-2
CD36: Fatty acid translocase
CPT-1A: Carnitine palmitoyl transferase 1A
CYP1A1: Cytochrome P450 family 1 subfamily A member 1
FABP-1: Fatty acid binding protein 1
FAS: Fatty acid synthase
FCR: Feed conversion rate
FLHS: Fatty liver hemorrhagic syndrome
FMT: Fecal microbiota transplantation
HDL-C: High-density lipoprotein cholesterol
H&E: Hematoxylin and eosin
HSL: Hormone-sensitive lipase
IAA: Indole-3-acctate
IAM: Indole-3-acctamide
IAAld: Indole-3-acctaldehyde
IL-6: Interleukin-6
IL-10: Interleukin-10
LDL-C: Low density lipoprotein cholesterol
LEfSe: Linear discriminant analysis effect size
LPL: Lipoprotein lipase
LR: Laying rate
MAG: Magnolol
MUC-2: Mucin-2
NF-κB: Nuclear factor kappa B
OUT: Operational taxonomic unit
PCA: Principal component analysis
PCoA: Principal coordinate analysis
PLS-DA: Partial least squares discriminant analysis
PPARα: Peroxisome proliferator activated receptor alpha
PPARγ: Peroxisome proliferator activated receptor gamma
SCD: Stearoyl-CoA desaturase
TC: Total cholesterol
TEM: Transmission electron microscope
TG: Triglyceride
TNF-α: Tumor necrosis factor-α
TRP: L-Tryptophan
TRM: Tryptamine
ZO-1: Zonula occludens-1
5-HAA: 5-Hydroxyindoleacetic acid
