# Supplementary material for: Hepatoprotective effects of magnolol in fatty liver hemorrhagic syndrome hens through shaping gut microbiota and tryptophan metabolic profile
Source: J Anim Sci Biotechnol. 2024 Sep 6;15:120. doi: 10.1186/s40104-024-01074-9 (PMC11378483; doi:10.1186/s40104-024-01074-9)
Supplement: Supplementary file 1 — Additional file 1: Table S1. Sequences of real-time PCR primers. [file 40104_2024_1074_MOESM1_ESM.docx]

**Table S1** The primers for qRT-PCR assays

| **Target genes** | **Forward primer (5′→3′)** | **Reverse primer (5′→3′)** | **GenBank accession** | **Products size, bp** |
| --- | --- | --- | --- | --- |
| *β-actin* | GATATTGCTGCGCTCGTTGT | TACCAACCATCACACCCTGAT | NM_205518.2 | 130 |
| *ACACα* | TTGTGGCACAGAAGAGGGAA | GCTCCAGATGGCGGTAGATT | NM_205505.2 | 90 |
| *FAS* | TGACAAAGCACTCGGTTTGG | TGACTCGCAATGTTCACACC | NM_001199487.2 | 115 |
| *SCD* | GGTACGCAAACACCCAGATG | TGAGGGCTTGTAGTATCTCCG | NM_204890.2 | 106 |
| *CD36* | TTGGTTAATGGCACTGATGGG | TCACGGTCTTACTGGTCTGGT | NM_001030731.1 | 124 |
| *HSL* | TTGCTTCCGAAGAATCCGGG | ATGGATGGCACGAACTGGAA | XM_040657096.1 | 109 |
| *LPL* | TGGACAGATGGACAGCTTGG | TCATACATTCCTGTCACCGTC | NM_205282.2 | 93 |
| *FABP-1* | GGAGAGAAGGCCAAGTGTATT | TAGGTGAGGTCTCCCTTCGT | NM_204192.4 | 134 |
| *CPT-1A* | TCAGACACCACAGCAACACA | CATCATCAGCCACAGGTCCA | NM_001012898.1 | 86 |
| *ACOX1* | CGAAAGGAGATCGAGGCCTTA | GCTTGTTCATAGCGTTGGCT | NM_001006205.2 | 92 |
| *PPARα* | TTCAATGCACTGGAACTGGA | AGACCAGGACGATCTCCACA | NM_001001464.1 | 80 |
| *PPARγ* | AGACGACAGACAAATCACCAT | AGAGCGAAACTGACATCGCT | NM_001001460.2 | 149 |
| Claudin-1 | TACTCCTGGGTCTGGTTGGT | GTGCTGACAGACCTGCAATG | NM_001013611.2 | 138 |
| Claudin-5 | TGTCAGCCTTCATCGACGTG | TGGAATCGTACACCTTGCACT | NM_204201.2 | 114 |
| Occludin | TCATCGTCATCCTGCTCTGC | CACGTTCTTCACCCACTCCT | NM_205128.1 | 146 |
| *ZO-1* | AACGCAGCTATTATCCGGCA | ATGCTCATAGCGAGGTCTGC | XM_040706827.2 | 80 |
| *AhR* | GCCAAAACACAGGAGATGGC | GGGAGGCACGGAGAAATGAA | XM_046917049.1 | 107 |
| *CYP1A1* | AGAAGCAGAGCTTCAACCCC | GGTGTTCATGTTCACCACGC | NM_205147.2 | 128 |
| *MUC-2* | GCTCACCCTGCATGGATACT | TCATACACAGTCCCTTCGGG | XM_040701654.2 | 100 |
| *NF-κB* | TGATCCAGCAGATGGACCGT | AGAAGCACCAGGAAGTCCAC | XM_046939919.1 | 112 |
| *TNF-α* | GATCGTGACACGTCTCTGCT | AACCAGCTATGCACCCCAG | NM_204267.2 | 86 |
| *IL-6* | AACAACCTCAACCTGCCCAA | TTCGTCAGGCATTTCTCCTCG | NM_204628.2 | 80 |
| *IL-1β* | CTGCCTGCAGAAGAAGCCT | TCCGCAGCAGTTTGGTCAT | NM_204524.2 | 137 |
| *Bax* | AACCCCAGCATTATCCCCAC | GGAGAGCAGAAAACCTCCCA | XM_040654813.2 | 120 |
| *Bcl-2* | CAACATTGCCACCTGGATGAC | CACAAAGGCATCCCATCCTC | NM_205339.3 | 85 |
| Caspase 3 | TGAAGGAACACGCCAGGAAA | GTCGAGTGGAGCAGGATTCA | NM_204725.2 | 90 |
| Caspase 8 | TGTGGCAAAGTGGACAAGAG | AGCCAAATGGCACTGTCTTC | NM_204592.4 | 134 |
